# Editorial: Exploring life skills and positive youth development through sports

**DOI:** 10.3389/fspor.2025.1725678

**Published:** 2026-01-06

**Authors:** Antonio Muñoz-Llerena, Elena Hernández-Hernández, Luís Murta, Jose Omar Lagunes-Carrasco

**Affiliations:** 1 Physical Education and Sports Department, Sevilla University, Seville, Spain; 2 Sports and Computers Department, Universidad Pablo de Olavide, Seville, Spain; 3 Instituto Politecnico de Beja, Beja, Portugal; 4 Universidad Autonoma de Nuevo Leon Facultad de Organizacion Deportiva, San Nicolás de los Garza, Mexico

**Keywords:** life skills, positive youth development, sport, transference, young people

Positive Youth Development (PYD) has become a cornerstone paradigm in youth sports research, shifting the focus from deficit prevention to proactively building strengths and cultivating supportive relationships. Over the past two decades, research have documented how intentionally designed sports programs cultivate what we know as life skills, this is, transferable competencies that enable young people to navigate challenges and seize opportunities both within and beyond the sports field. This Research Topic, “*Exploring Life Skills and Positive Youth Development through Sports*,” builds on this foundation, aiming to deepen understanding of both theoretical underpinnings and practical applications of PYD programs in fostering life skills development. The six contributions to this Research Topic offer diverse and valuable insights, examining this topic across varied cultural contexts, competitive levels, educational settings, and social ecosystems.

A central theme emerging from this collection is the pivotal role of social agents, especially coaches, in shaping young athletes' developmental experiences. Berntzen and Lagestad provide robust experimental evidence from Norwegian youth football that coach feedback serves as a powerful tool for enhancing psychological well-being. Their intervention revealed that structured feedback enhanced athletes' sense of mastery, motivation, and perception of coach acknowledgment. These benefits proved particularly pronounced among athletes in specialized programs, who also reported greater well-being, pleasure, and satisfaction. This is a finding that underscores the value of context-specific feedback approaches. Building on this, Nakayama and Izawa examined transformational leadership among coaches in the unique cultural context of Japanese youth sports. They found strong associations between coaches' high-performance expectations and athletes' goal-setting and initiative; these relationships were more pronounced than those documented in Western contexts. Their findings illustrate how deeply cultural context can mediate the relationship between coaching behaviors and PYD outcomes, suggesting that high expectations, while motivating, must be balanced with supportive leadership in order to achieve holistic development.

The developmental context represents another critical dimension explored within this Research Topic. Hoare et al. examined a multi-component mental wellbeing program for adolescent athletes in a high-performance Australian Rules Football talent pathway. Their qualitative analysis revealed gradual acceptance and perceived value of the intervention program, which was based on the PERMA model of well-being, while emphasizing the importance of strengthening support capacity across the entire football community. By contrast, Crowther et al. examined a community-based initiative, the “JU:MP leads” program, implemented in a deprived United Kingdom neighborhood. This program successfully developed young people into physical activity leaders, fostering life skills such as leadership, communication, and confidence. The “JU:MP leads” program's success could be explained due to its flexible delivery, strong mentorship structures, and perhaps most powerfully, its relatability: young leaders served as authentic role models, especially for peers from similar ethnic backgrounds. Taken together, these two studies demonstrate that PYD can flourish across settings, from elite or high performance pathways to grassroots programs, when approaches are contextually adapted and adequately supported.

Moving beyond specific sports programs, two additional studies examined life skills development in educational and international contexts. Madrid-Rísquez et al. conducted a cross-sectional analysis of students in a Spanish vocational training program for outdoor activity guidance, examining their cognitive profiles. They found considerable variability in students' IQ and cognitive life skills, such as verbal comprehension and processing speed. Their results pointed to a clear need for individualized educational support in developing cognitive capacities essential for academic and professional success. In a complementary longitudinal approach, Nyanyo Nyanyofio et al. provided a a long-term perspective by assessing the sustained impact of the Africa Alliance for Partnerships program in Ghana on its alumni five years post-completion. They documented significant, sustained improvements in entrepreneurial skills, resilience, leadership, and teamwork, which positively influenced participants' academic and career trajectories. Their work offers compelling evidence that sports-based development programs can function as sustainable ways for entrepreneurship and personal growth, even where resources are scarce.

Collectively, these six contributions enrich our understanding of how sports can be a powerful vehicle for PYD. Several themes emerge across the collection. First is intentionality: whether in the form of targeted coach feedback or systematic program design, deliberate action matters. Second is the critical importance of context: effective strategies require adaptation to cultural norms, competitive levels, and community characteristics and needs. Third, the studies connect life skills development to concrete outcomes, from psychological well-being to academic success and long-term career progression. Methodologically, the collection's diversity (ranging from quantitative experimental designs and large-scale surveys to qualitative case studies) provide a robust and multi-faceted evidence base.

In conclusion, this Research Topic paves the way for future research, highlighing the need for longitudinal research focused on how life skills transfer across time and life domains. We thank the authors, reviewers, and Frontiers editorial team for their invaluable contributions to this Research Topic. Together, they have produced a body of work that will undoubtedly inspire further research and inform practice in our collective efforts to to help young people thrive through sport.

